# Microstructural imaging and transcriptomics of the basal forebrain in first-episode psychosis

**DOI:** 10.1038/s41398-022-02136-0

**Published:** 2022-09-01

**Authors:** Min Tae M. Park, Peter Jeon, Leon French, Kara Dempster, M. Mallar Chakravarty, Michael MacKinley, Julie Richard, Ali R. Khan, Jean Théberge, Lena Palaniyappan

**Affiliations:** 1 Department of Psychiatry, Schulich School of Medicine and Dentistry, Western University, London, Canada; 2 Department of Medical Biophysics, Western University, London, Canada; 3 Robarts Research Institute, Western University, London, Canada; 4 Lawson Health Research Institute, London, Canada; 5 Department of Psychiatry, University of Toronto, Toronto, Canada; 6 Department of Psychiatry, Dalhousie University, Halifax, Canada; 7 Departments of Psychiatry and Biological and Biomedical Engineering, McGill University, Montreal, Canada; 8 Cerebral Imaging Centre, Douglas Research Centre, Montreal, Canada; 9 Douglas Mental Health University Institute, Department of Psychiatry, McGill University, Montreal, Canada

**Keywords:** Schizophrenia, Prognostic markers

## Abstract

Cholinergic dysfunction has been implicated in the pathophysiology of psychosis and psychiatric disorders such as schizophrenia, depression, and bipolar disorder. The basal forebrain (BF) cholinergic nuclei, defined as cholinergic cell groups Ch1-3 and Ch4 (Nucleus Basalis of Meynert; NBM), provide extensive cholinergic projections to the rest of the brain. Here, we examined microstructural neuroimaging measures of the cholinergic nuclei in patients with untreated psychosis (~31 weeks of psychosis, <2 defined daily dose of antipsychotics) and used magnetic resonance spectroscopy (MRS) and transcriptomic data to support our findings. We used a cytoarchitectonic atlas of the BF to map the nuclei and obtained measures of myelin (quantitative T1, or qT1 as myelin surrogate) and microstructure (axial diffusion; AxD). In a clinical sample (*n* = 85; 29 healthy controls, 56 first-episode psychosis), we found significant correlations between qT1 of Ch1-3, left NBM and MRS-based dorsal anterior cingulate choline in healthy controls while this relationship was disrupted in FEP (*p* > 0.05). Case-control differences in qT1 and AxD were observed in the Ch1-3, with increased qT1 (reflecting reduced myelin content) and AxD (reflecting reduced axonal integrity). We found clinical correlates between left NBM qT1 with manic symptom severity, and AxD with negative symptom burden in FEP. Intracortical and subcortical myelin maps were derived and correlated with BF myelin. BF-cortical and BF-subcortical myelin correlations demonstrate known projection patterns from the BF. Using data from the Allen Human Brain Atlas, cholinergic nuclei showed significant enrichment for schizophrenia and depression-related genes. Cell-type specific enrichment indicated enrichment for cholinergic neuron markers as expected. Further relating the neuroimaging correlations to transcriptomics demonstrated links with cholinergic receptor genes and cell type markers of oligodendrocytes and cholinergic neurons, providing biological validity to the measures. These results provide genetic, neuroimaging, and clinical evidence for cholinergic dysfunction in schizophrenia.

## Introduction

Dysregulation of the cholinergic system has been long suspected in the pathophysiology of psychotic disorders such as schizophrenia [1, 2]. Several lines of evidence support a cholinergic imbalance in psychosis [3], particularly in the negative symptoms such as psychomotor retardation and inattention [4, 5]. The cholinergic hyperactivity induced by physostigmine worsens negative symptoms [4], while anticholinergics provide some relief for negative symptoms [6, 7]. Muscarinic (M1/M4) agonist xanomeline has been shown to have clinical efficacy in schizophrenia [8, 9]. Post-mortem human studies have identified a biological basis for these observations; specifically, the distributed reduction of muscarinic receptors in a subset of patients [10, 11]. Taken together, these findings motivate calls for focused investigations of the cholinergic system to aid therapeutic discoveries in psychosis [9].

Despite the substantial evidence for a cholinergic abnormality in schizophrenia, it is not clear how a disrupted cholinergic system relates to the first presentation of psychosis, before treatments with anticholinergic effects are started. The three major hurdles in this regard are (1) the challenges in non-invasive study of the basal forebrain (BF), a structure that provides cholinergic projections to extensive areas of the cortical mantle; being a deep brain structure, both direct non-invasive stimulation and surface level recordings of activity are difficult to obtain, (2) the difficulties in direct quantification of acetylcholine [12] through in vivo imaging studies as field inhomogeneities of the basal brain regions limit neurochemical imaging and acetylcholine is not readily separable from other choline-containing compounds, (3) challenges in studying symptomatic untreated patients without the confounds of illness chronicity and long term antipsychotic exposure [13], as many acutely unwell patients do not prefer prolonged MRI sessions.

There are a number of whole brain morphometric studies [14], and a region-of-interest study [15] of BF in schizophrenia, yet none have identified notable changes in the basal forebrain. A recent study specifically assessed grey matter volume of the BF in schizophrenia, while results were not significant after taking into account global effects [16]. In a focused post-mortem examination of Nucleus Basalis of Meynert (NBM), Williams and colleagues [17] identified a notable reduction in oligodendrocyte density and glial cell abnormalities in schizophrenia. It is unknown if these changes are restricted to the NBM, which primarily projects to the neocortex, or if they extend to anteromedial BF nuclei (Ch1-3, or Broca’s diagonal band) that project to the hippocampus [18]. The vulnerability of BF has been linked to its later onset of myelination and the relative sparsity of myelin sheaths in the BF compared to other regions of the brain [19–21]. Higher myelin concentration may reflect reduced metabolic demands on the ensheathed neurons, and thus higher resilience to degenerative processes [22].

The relative lack of grey-white matter differentiation in the basal forebrain often limits accurate volumetric measurements, yet advances in human neuroimaging have provided us with probabilistic maps of BF based on cytoarchitectonic studies [23]. Though oligodendrocytes cannot be directly measured using MRI in humans, a number of related microstructural properties can be examined in vivo. Of particular relevance is quantitative T1 or qT1 which has a high negative correlation with myelin content [24, 25], while axial diffusivity (AxD) best captures axonal injury [26]; both qT1 and AxD increase in experimental animal models of demyelination [27].

While microstructural changes of the basal forebrain may be present across several disorders, it is unclear whether this structural variation translates differences in acetylcholine levels and cholinergic tone in the cortex. In humans, acetylcholine levels could be estimated using magnetic resonance spectroscopy (MRS), which may aid the interpretation of microstructural findings. Acetylcholine and free choline, the precursor of acetylcholine, together contribute to a small portion of the MRS total choline spectra, but this signal is not separable from that of other membrane bound choline moieties [28]. Nevertheless, several observations suggest that the variations in the MRS choline signal may reflect variations in cholinergic tone. Xanomeline, a muscarinic agonist, decreases MRS choline resonance in Alzheimer’s Disease patients [29]. In mice, the anticholinergic scopolamine induces an acute reduction in MRS choline signal that returns to baseline in 72 h [30], while anticholinesterase donepezil increases choline resonance [31]. In rats, MRS choline signal intensity shows a high degree of correlation with direct tissue measurement of acetylcholine levels across various brain regions [32]. In humans, free choline, when teased apart from bound choline, changes with the performance of tasks such as reversal learning that are dependent upon cholinergic transmission [28, 33]. While these fMRS studies have focussed on striatal choline signal, the diffuse prefrontal cholinergic projections from the basal forebrain [34–36], indicate that the overall cholinergic tone of BF projection are best estimated from the frontal cortical regions. MRS choline is increasingly being used as a proxy measure for acetylcholine levels in the anterior cingulate cortex (ACC) in patients with psychosis [37].

The ACC is an important site of cholinergic projection from the basal forebrain in animals [38] and humans [18, 39]. In particular, the NBM is specifically connected to the dorsal ACC (dACC) component of the Salience Network [18] (SN), enabling contextual integration and cognitive control function [40]. The SN, in turn, plays a critical role in the resource allocation for stimulus evaluation and action outcomes that involve the deployment of large-scale cortical networks [41]. Prior research, from our groups and others [42–45], has implicated the SN in the pathophysiology of psychosis. Given the cholinergic hypothesis of psychosis, and the SN dysfunction observed in this illness, it is likely that the structural integrity of basal forebrain cholinergic nuclei influences the cholinergic tone of the SN, but this has not been evaluated to date.

Neuroimaging studies may identify broad changes in brain structure and function, but with limitations in identifying genetic and cellular perturbations. Large-scale datasets such as the Allen Human Brain Atlas (AHBA [46]) allow for anatomically comprehensive studies of fine-grained structures [47, 48]. Previous transcriptomic studies of the BF have focused on conditions such as traumatic encephalopathy [49] and dementia [50], with no past gene expression studies of the BF in humans or in relation to schizophrenia. In addition, recent work bridging neuroimaging and transcriptomics supported the biological basis for neuroimaging correlations [51], further increasing the value of gene expression analyses in complementing neuroimaging data.

Here, we examine the microstructure of the basal forebrain cholinergic nuclei in first-episode psychosis (FEP) using ultra-high resolution imaging. We quantified choline resonance from the dACC using 7T proton MRS. We related this resonance to BF qT1 in 56 patients with first episode psychosis and 29 healthy individuals, anticipating a dissociation between the microstructure of BF and dACC choline levels in patients. Given the reported reduction in oligodendrocyte density in schizophrenia [17], we expected the microstructural changes of increased qT1 and AxD in patients with untreated first episode psychosis.

Furthermore, on the basis of Tandon’s hypothesis that higher cholinergic tone underlies greater negative symptoms [4, 6, 7, 52, 53], Janowsky’s cholinergic hypothesis of mania [54, 55] that implies reduced cholinergic tone predict higher manic symptoms, and given past evidence of ACHe inhibitors improving manic symptoms [56], we tested if higher qT1 (low myelin) and thus reduced cholinergic tone would correlate with greater manic symptom severity, and lower qT1 (high myelin) and increased cholinergic tone to predict greater negative symptom burden.

We tested covariance between qT1 of BF and cortical-hippocampal regions to test the biological validity of BF qT1. This is based on previous work that shows cellular similarity, and connectivity as a basis for correlations between morphometric measures [51]. We hypothesised that the most significant correlations would reflect (1) Brain regions receiving cholinergic input from the basal forebrain, and (2) Brain regions with relatively higher cholinergic neuron content. In the hippocampus, we expect CA1-subiculum to demonstrate strongest correlations given it has a higher density of cholinergic neurons based on choline aceyltransferase (ChAT) expression [57], and both CA1 and subiculum receives projections from the BF [58]. In the cortex, we expect strongest correlations with the frontal, entorhinal, and anterior cingulate cortices based on tracer studies [59–61], and also frontal, entorhinal, and motor/sensory regions [34].

Through transcriptomic analyses, we expected gene expression of the basal forebrain to show enrichment for schizophrenia-related genes given previous links between psychiatric disorders and cholinergic dysfunction. In imaging-transcriptomic correlations of BF-cortical covariance, we expect enrichment for oligodendrocyte marker genes given qT1 measures myelin content and qT1-based correlations would be driven by myelin-related genes. We also expect enrichment for cholinergic neurons given presence of cholinergic interneurons may also drive correlations between the cortex and BF. Amongst the genes, we expect significant associations with cholinergic receptor genes as regions with high correlations are more likely to receive cholinergic input from the BF.

## Materials and methods

### Clinical participant recruitment and assessment

Details regarding recruitment have been described in our previous work [44, 62, 63] but included here for completion. Participants were recruited as part of a neuroimaging project tracking changes in early psychosis from the PEPP (Prevention and Early Intervention for Psychosis Programme) at London Health Sciences Centre. All participants provided written, informed consent with approval from Western University Health Sciences Research Ethics Board. Inclusion criteria were as follows: individuals experiencing FEP, with lifetime antipsychotic treatment less than 14 days. Exclusion criteria for FEP included: meeting criteria for a mood disorder (bipolar or major depressive) with psychotic features, or possible drug-induced psychosis. Healthy control (HC) participants were free from personal history of mental illness or family history of psychotic disorders, matched based on age, sex, and parental education. Exclusion criteria for both FEP and HC included substance use disorder in the past year based on DSM-5 criteria, history of major head injury, significant medical illness, or contraindications to MRI.

Participants were assessed using DSM-5 criteria and the 8 item Positive and Negative Syndrome Scale (PANSS-8), Young Mania Rating Scale (YMRS), the Calgary Depression Scale (CDS), and cannabis use evaluated using the Cannabis Abuse Screening Test (CAST). Parental socioeconomic status (SES) was quantified on the National Statistics Socio-economic classification (NS-SEC), based on the parent’s highest occupation in the past 5 years. Cognitive tests included digit symbol substitution test (DSST—average of written and oral), and trail making test. Substance use in the past year was assessed based on self-report questionnaire for healthy controls, and self-report, DSM-based clinical assessments, and urine drug screening at time of clinic entry (not at time of scanning) in suspected cases for patients.

### Acquisition of neuroimaging and preprocessing

Details regarding imaging acquisition parameters and preprocessing are provided in Supplementary Information—briefly, we acquired T1-weighted images (qT1), diffusion tensor imaging, and MRS measures of choline in the dACC. Voxel size for structural imaging (T1) was 0.8 mm × 0.8 mm × 0.8 mm, while for DTI it was 2 mm × 2 mm × 2 mm. MRS voxel measuring 2.0 cm × 2.0 cm × 2.0 cm (8 cm^3^) was placed in the bilateral dACC.

Preprocessing for T1-weighted images are in line with our previous work [63], as well as DTI [64] and MRS [62, 63]. Following preprocessing, we used the T1-weighted images to map the cortex and subcortical structures using automated methods (see Supplementary Methods). The basal forebrain is defined using a published probabilistic atlas based on cytoarchitectonic mapping of cholinergic cell groups, Ch1-3 and Ch4 (NBM) [23], and warped to individual subject T1-weighted images using ANTs [65] (Supplementary Methods).

### MRS and MRS-structural analyses

We first compared MRS choline between HC and FEP using multiple linear regression, controlling for age, sex, smoking status, and cannabis use. We then tested for correlations between dACC choline and BF measures separately in HC and FEP, expecting significant correlations in HC but not in FEP. We expected BF measures to predict cholinergic tone, and in HC, we expected qT1 to correlate negatively with choline—such that lower qT1 (high myelin) would reflect higher cholinergic tone and elevated levels of choline.

We tested whether the BF-choline correlations were significantly different between groups by modelling the diagnosis-by-qT1 interaction effect on choline levels, accounting for age and sex as covariates. Smoking status and cannabis use were excluded in interaction analysis since the healthy control group had no smokers, and the FEP group had significantly higher cannabis use (*t* = 5.63, *p* = 4.50E−07, DF = 64). Threshold of significance is based on multiple testing correction for 3 comparisons (left, right NBM, and Ch1-3 with dACC choline) with *p*_Bonferroni_ < 0.017.

### Structural neuroimaging analyses

In case-control analysis, we compared neuroimaging measures of BF (qT1, AxD) between HC and FEP, using multiple linear regression seeking the main effect of diagnosis accounting for age, sex, cannabis use (CAST), and smoking status as covariates. Multiple testing correction is based on 3 comparisons (left, right NBM and Ch1-3) between groups with *p*_Bonf_ < 0.017 deemed as significant. We tested for correlations between left NBM and Ch1-3 (qT1 and AxD) with clinical measures of total YMRS and PANSS-8 negative symptom scores. Multiple testing correction is based on 8 tests (2 structures × 2 metrics × 2 clinical scores) with *p*_Bonf_ threshold < 6.25E−03.

We correlated qT1 values between the BF and the rest of the brain including the subcortical brain structures (hippocampus, amygdala, striatum, thalamus, globus pallidum) and cortex. Pearson’s R correlations were corrected for multiple testing by FDR within each structure, with significance threshold of FDR *q* < 0.10.

### Transcriptomic analysis of basal forebrain

We examined gene expression profiles of the BF structures to determine whether transcriptomics would reveal enrichment for cholinergic cell type markers, and disease associations. We used gene expression data from the Allen Human Brain Atlas [46]. Quality control was done by visual examination of BF samples on the MNI template. One Ch1-3 sample was found to be misplaced or mislabelled (see Supplementary Fig. 1), and therefore excluded from analysis. For the NBM, 4 donors contributed 9 NBM samples and the same 4 donors contributed 8 Ch1-3 samples (nucleus of the diagonal band, 4 horizontal and 4 vertical division) after quality control.

Probe selection was based on published quality control metrics based on comparison between the Agilent microarray and “ground truth” RNA-Seq [66]. We selected those probes that passed quality control and selected one probe per gene based on highest correlation to RNA-seq data. Probe-to-gene mappings were provided by the Allen Institute.

We identified genes that were specifically expressed in the Ch1-3 and NBM. Region-specific expression analysis, as in previous studies [48, 67], was done using R (version 4.0) and the limma package to detect genes specifically expressed in the BF. For this analysis, all samples are included, restricted to only those donors that sampled the Ch1-3 and NBM.

For each gene, linear models were fit to gene expression with terms for the donor and region of interest. Moderated t statistics and p-values were calculated using the empirical Bayes moderation method, and corrected for multiple comparisons using Benjamini–Hochberg false discovery rate (FDR) [68]. Genes were ranked based on significance (−log10 of *p*-value) multiplied by the sign of the t-statistic, and we selected the most significantly enriched genes after FDR correction at *q* < 0.10. These gene lists were submitted to the cell-type specific expression analysis (CSEA), which we expect to show enrichment for cholinergic neurons [69, 70]. ToppGene [71] was used to test for disease enrichment (at *q* < 0.10), using the DisGeNet Curated database, exploring whether there was enrichment for schizophrenia-related genes.

### Imaging-transcriptomic validation

We sought to explain BF-cortical imaging correlations with gene expression. We used data from the AHBA for the left cortex only since only 2 of the 6 AHBA brains sampled the right hemisphere. Only cortical samples are retained (samples from non-cortical, i.e., subcortical regions are removed), prior to finding a 1-to-1 match between each AHBA cortical sample and CIVET-generated vertex (Supplementary Fig. 2a).

For matching AHBA samples to vertices on the CIVET cortical template, we used the set of re-registered AHBA sample coordinates to the MNI ICBM 2009c (nonlinear symmetric) template (generated by Gabriel A. Devenyi; https://github.com/gdevenyi/AllenHumanGeneMNI). This improves anatomical concordance between the original anatomical sample and MRI using multispectral and non-linear registration, as the original AHBA-provided MNI coordinates were calculated using an affine registration only. The ICBM template is also processed through CIVET 2.1.0 to generate a cortical surface. We assigned each AHBA sample to the closest vertex based on Euclidean distance. Where multiple samples matched to the same vertex, the closest sample in terms of distance was retained. We removed 2 samples with distance greater than 10 mm, which were in the right hemisphere based on visual inspection (Supplementary Fig. 2b). We matched 1236 AHBA samples to a unique vertex, with mean distance of 1.47 between sample and vertex (sd 0.89, range 0.084–6.78 mm).

Pearson’s R correlations for the left cortex (as described above) were modelled using mixed effects models, with gene expression as the fixed and donor as random effect. This is repeated across ~14,000 genes and resulting *p*-values are corrected using FDR. Top and bottom 10% of ranked genes were submitted to CSEA.

### Post hoc analyses

We explored the relationship between cognitive scores (DSST, trail making test) and neuroimaging measures (qT1 and AxD). We also examined radial diffusivity (RD) as an alternative measure for myelin, in relating to MRS choline, case-control differences, and clinical scores.

## Results

### Sample characteristics

After quality control of neuroimaging measures, 56 FEP and 29 HC were available for analysis. There were no significant differences between groups in terms of age or sex distribution (Table 1). Cognitive scores were significantly different between groups, with lower DSST scores and greater trail making test times in FEP compared to HC (Table 1).

**Table 1:** Summary of sample demographics and clinical characteristics.

	FEP (*N* = 56)	HC (*N* = 29)	*t*/*Χ*^2^	*P*-value
Age	22.34 (4.30)	21.72 (3.46)	0.67	0.507
Sex (male/female)	43/13	18/11	1.38	0.24
Parental SES	3.49 (1.23)	3.21 (1.45)	0.89	0.38
PANSS-8 total	25.24 (6.62)			
Total positive	12.60 (2.59)			
Total negative	7.17 (4.26)			
Total CDS	3.67 (3.31)			
SOFAS	39.67 (12.94)			
Mean DSST (sd)	51.77 (13.97)	68.71 (11.30)	−5.62	2.51E−07
Mean trail making test (sd)	88.34 (74.86)	54.88 (15.26)	2.20	0.032
Mean DUP (sd) (weeks)	31.13 (56.62)			
Mean DDD (sd) (days)	1.32 (2.10)			
Antidepressants at time of scanning	5			
Consensus diagnosis	2 MDD			
	2 Bipolar			
	1 Clinical High Risk			
	3 Schizoaffective			
	1 Schizophreniform			
	44 Schizophrenia			
	3 Psychosis NOS			

*CDS* Calgary Depression Scale, *SOFAS* Social and Occupational Functioning Assessment Scale, *DUP* duration of untreated psychosis, *DDD* defined daily dose (lifetime).

### MRS-structural correlations

We tested for correlations between structural measures and dACC choline. There was no significant difference in dACC choline between HC and FEP (*t* = 1.08, *p* = 0.284, DF = 78) after controlling for age, sex, smoking status and cannabis use. Mean CRLB of choline was 1.88 (sd 0.52, range 1.18–3.58).

**Fig. 1:**
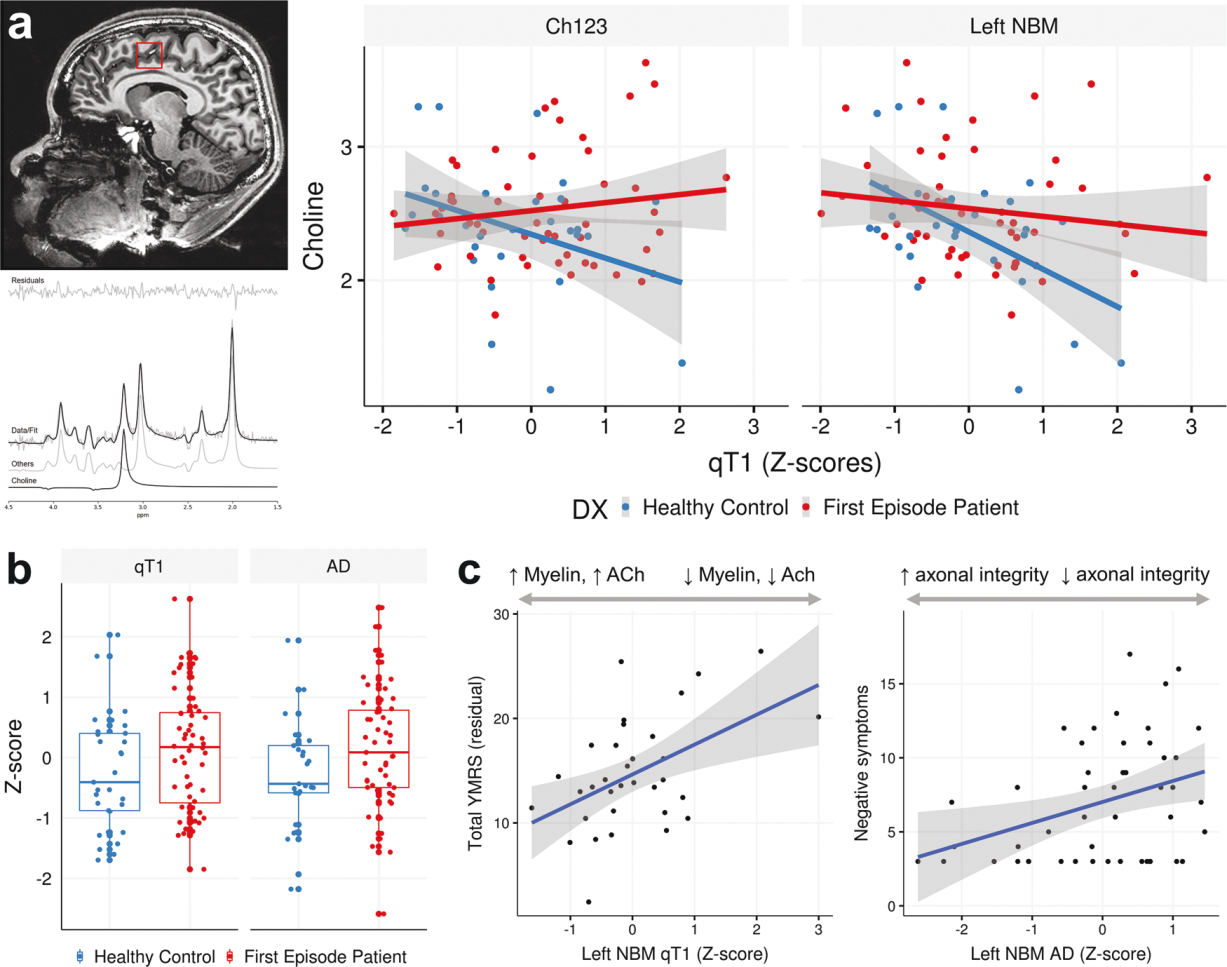
Evidence for microstructural changes of the basal forebrain in FEP. **a** Left: Magnetic resonance spectroscopy of choline in the dACC with example of voxel placement (top), and spectral fit for choline (bottom). Right: In FEP, there is decoupling of the correlation between qT1 and choline levels, while a significant correlation exists in healthy controls such that lower qT1 (higher myelin) is associated with elevated choline levels. **b** In the Ch1-3, there is increased qT1 (lower myelin), and increased axial diffusivity. **c** Higher qT1 (indicating lower myelin) of the left NBM is associated with greater manic symptom severity, and increased AxD (reflecting lower axonal integrity) is associated with negative symptom severity. Left: *y*-axis shows residuals of the linear regression (YMRS ~ cannabis use) added to the mean YMRS score.

In healthy controls (*N* = 29), we found significant correlations between dACC choline and left NBM qT1 (Pearson’s R = −0.517, *p* = 4.08E–03, DF = 27), marginally so for Ch1-3 (*R* = −0.35, *p* = 0.062), but not the right NBM (*R* = −0.238, *p* = 0.2127) (Fig. 1a). After accounting for influence of covariates including age, sex, and cannabis use on choline, correlations continued to be significant—for Ch1-3 (*t* = −2.62, *p* = 0.015, DF = 23), and left NBM (*t* = −4.34, *p* = 2.4E−04, DF = 23). These correlations were not significant in the FEP group (*p* = 0.292, 0.874, 0.307 for left, right NBM and Ch1-3 respectively).

We tested for the differences in slopes by modelling the diagnosis-by-qT1 interaction effect on choline levels which showed significant differences for Ch1-3 (*t* = 2.96, *p* = 4.14E−03, DF = 76), and left NBM (*t* = 2.72, *p* = 8.10E−03, DF = 76) after accounting for age, and sex as covariates (Fig. 1a).

### Case-control differences in BF microstructural measures

We found significant differences between HC and FEP in Ch1-3 qT1 (*t* = 2.717, *p* = 8.64E−03, DF = 59) after accounting for age, sex, cannabis use, and smoking as covariates (Fig. 1b). There were no differences in left or right NBM (*p* > 0.10). For the Ch1-3, mean AxD was increased in FEP (*t* = 2.90, *p* = 5.58E−03, DF = 50) with the same covariates (Fig. 1b). These results for Ch1-3 persist after including ICV as a covariate—for qT1 (*t* = 2.88, *p* = 5.57E−03, DF = 58) and AxD (*t* = 2.94, *p* = 5.02E−03, DF = 49). Overall, we found evidence for increased qT1 and AxD in the Ch1-3 (medial septum and diagonal band) of the basal forebrain in FEP, independent of brain size. Levene’s test was not significant (*p* > 0.05), demonstrating equal variance for all neuroimaging measures except for left Ch4 AxD (*F* = 6.73, *p* = 0.01).

### Basal forebrain microstructure in relation to clinical scores

There was a significant correlation between left NBM qT1 and YMRS (*t* = 3.03, *p* = 5.09E−03, DF = 29) after accounting for cannabis use and smoking status (Fig. 1c), accounting for 24.7% (adjusted R-squared) of the YMRS variance. Ch1-3 axial diffusivity correlated with YMRS neared significance (*R* = −0.286, *p* = 0.054), and less significant after accounting for cannabis use and smoking status (*p* = 0.90). Left NBM AxD was correlated with negative symptom severity (*R* = 0.358, *p* = 0.0115, DF = 47), remaining significant after accounting for smoking status and cannabis use (t = 2062, *p* = 0.0490, DF = 27). After multiple testing correction (2 structures × 2 metrics × 2 clinical scores = 8 tests), only the left NBM qT1 to YMRS correlation is significant (*p*_Bonf_ < 6.25E−03).

### Neuroimaging correlations

**Fig. 2:**
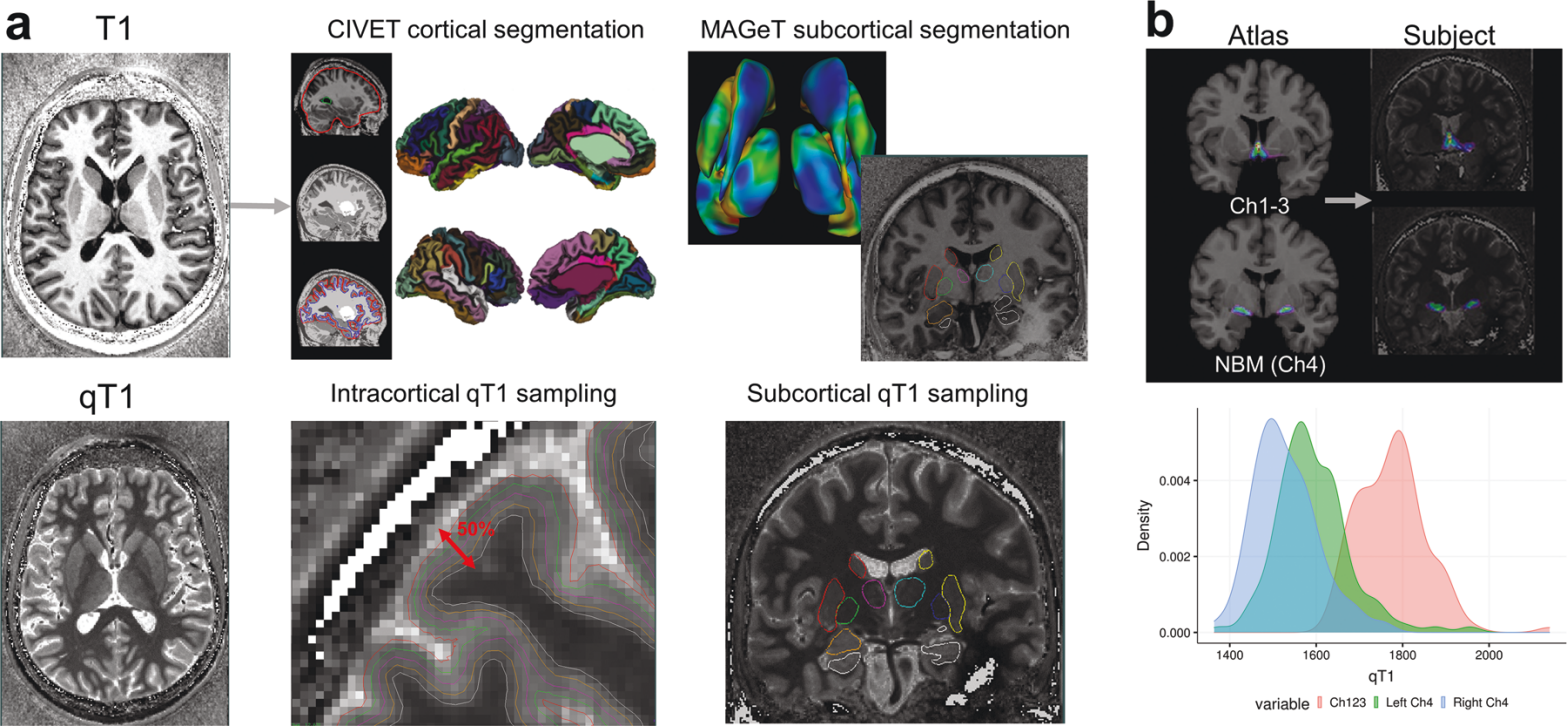
Image preprocessing of cortical, subcortical, and basal forebrain structures. **a** qT1 and T1-weighted MRI data at 7T were acquired using the MP2RAGE sequence. The CIVET pipeline (version 2.1.0) was used to delineate the cortical surfaces and sampling at multiple depths, and we sample qT1 measures at 50% depth from the pial to white matter surface. Subcortical qT1 sampling along MAGeT Brain-generated structures (hippocampus, amygdala, striatum, thalamus, globus pallidum). **b** Mapping probabilistic atlas based on cytoarchitectonic data of the basal forebrain structures onto individual subjects through non-linear registration. Histogram shows group distribution of qT1 values for the three structures—Ch1-3, left and right Ch4 (NBM).

Outlined in Fig. 2 is the neuroimaging preprocessing, including cortical and subcortical segmentation and sampling (Fig. 2a), registration of the probabilistic atlas to subject space (Fig. 2b, top) and resulting distribution of mean qT1 values as an example (Fig. 2b, bottom).

**Fig. 3:**
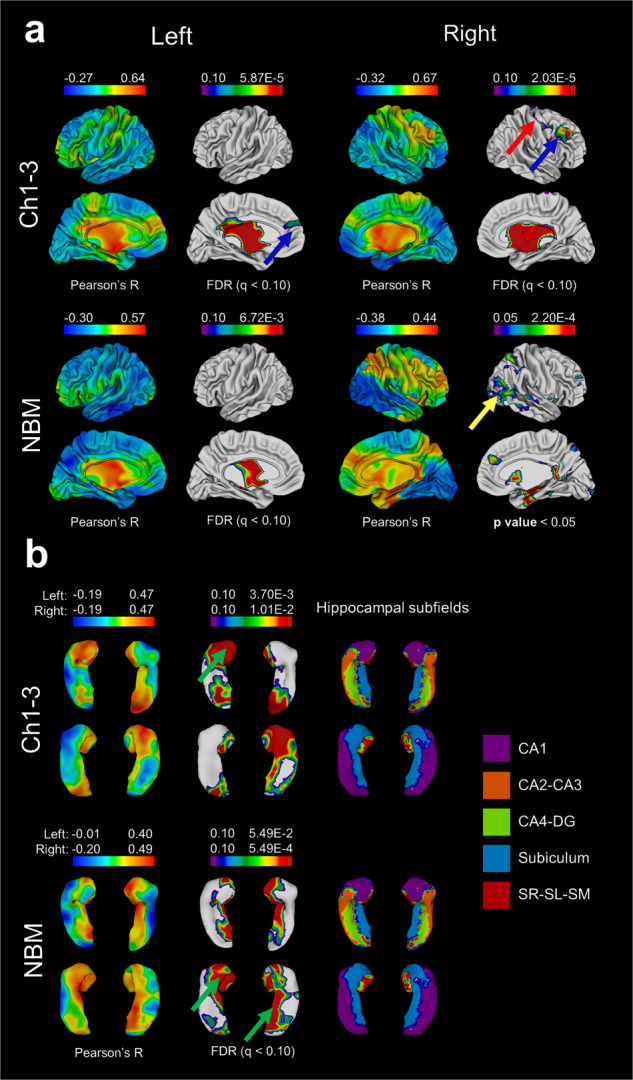
NBM-cortical and NBM-hippocampal qT1 correlations. Warm colours (red) indicate most positive correlations, and cool colours (blue) negative correlations for each set of surfaces. Regions surviving FDR correction are displayed, while for the right NBM-cortical correlations no regions survived FDR correction at *q* < 0.10, and thus subthreshold findings at *p* < 0.05 are shown instead. **a** NBM-cortical qT1 correlations. Findings are consistent with the hypothesis with strongest (most positive) correlations reflecting cortical regions receiving the most consistent afferent projections the BF, and those regions with cortical cholinergic neurons. These include the frontal (blue arrow), sensory/motor cortices (yellow arrow). **b** NBM-hippocampal qT1 correlations—for the hippocampus, CA1-subiculum demonstrate strongest correlations (green arrow).

Figure 3 shows the range of Pearson’s R values and thresholded (after FDR correction) regions from BF-cortical and BF-hippocampal qT1 correlations. For BF-cortical correlations, sample size was *N* = 65 (22 HC, 43 FEP) after quality control of CIVET cortical surfaces, and for BF-hippocampal correlations, *N* = 76 (27 HC, 49 FEP) after QC for subcortical segmentations.

Correlating qT1 values between the BF and cortex demonstrates the known projection pathways from the BF (Fig. 3a). The medial cortical surfaces show significant correlations, reflecting the medial pathway of Ch4 projections [72]. After FDR correction, we observed significant correlations for frontal (Fig. 3a, blue arrow), precentral/postcentral (red arrow). BF-hippocampal correlations show the CA1 and subiculum as the most prominent regions (Fig. 3b, green arrows).

### Transcriptomic analysis of BF structures

We examined genes with specific expression in the Ch1-3 and NBM, at FDR corrected thresholds of 10, 5, and 1%.

**Fig. 4:**
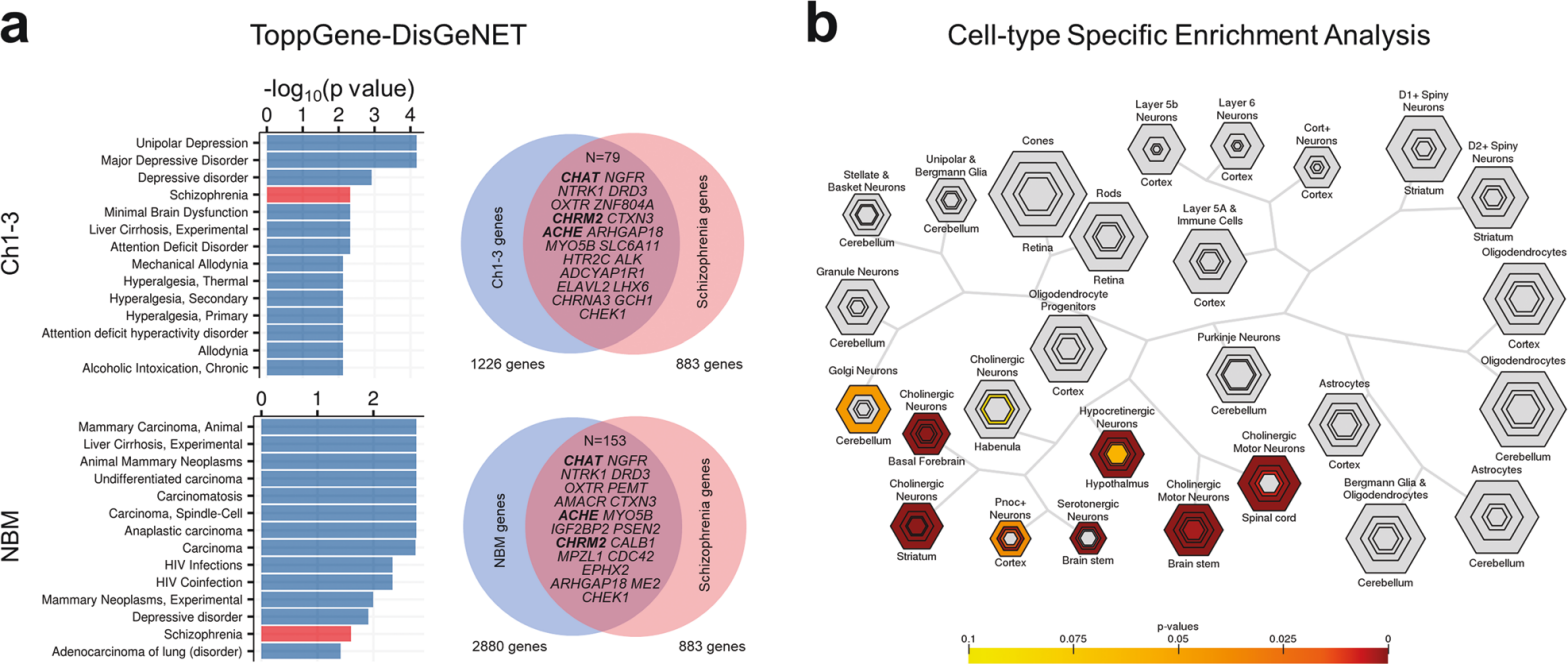
Transcriptomic analysis of the basal forebrain structures. **a** Enrichment of schizophrenia related genes. Venn diagram shows overlap between genes highly expressed in Ch1-3 and NBM with schizophrenia-related genes based on DisGeNET. Bolded genes are associated with cholinergic function (*CHAT, ACHE, CHRM2*). **b** CSEA of genes passing FDR (*q* < 0.10) shows enrichment for cholinergic neurons in the Ch1-3. Coloured cell types indicate significance after multiple testing correction (FDR *q* < 0.10). Hexagon sizes indicate cell-type specificity at varying specificity index probability (pSI) statistic thresholds from 0.05 to 1e−4, such that the outer hexagons indicate the least specific test for a cell type (pSI threshold 0.05) while the innermost hexagon indicates the most specific test for a cell type (pSI 1e−4).

ToppGene disease enrichment shows Ch1-3 (at FDR 10%) genes showing significant overlap with schizophrenia-related genes (1226 genes from Ch1-3, 79 genes overlapping with 883 schizophrenia-related genes) (*q* = 4.744E−03) (Fig. 4a). In the NBM, 2880 genes survived FDR 10%, and 153 genes overlapped (*q* = 2.51E−02). For both sets of overlapping genes, we found 4 acetylcholine-related genes including *CHAT, CHRM2, ACHE*, and *CHRNA3*. *CHAT* was the most significantly expressed gene in both Ch1-3 and NBM out of the intersecting genes, and *ACHE* was amongst the top 10 genes. CSEA showed enrichment for cholinergic neuron markers in the Ch1-3 and NBM (Fig. 4b).

### Imaging-transcriptomic analyses

**Fig. 5:**
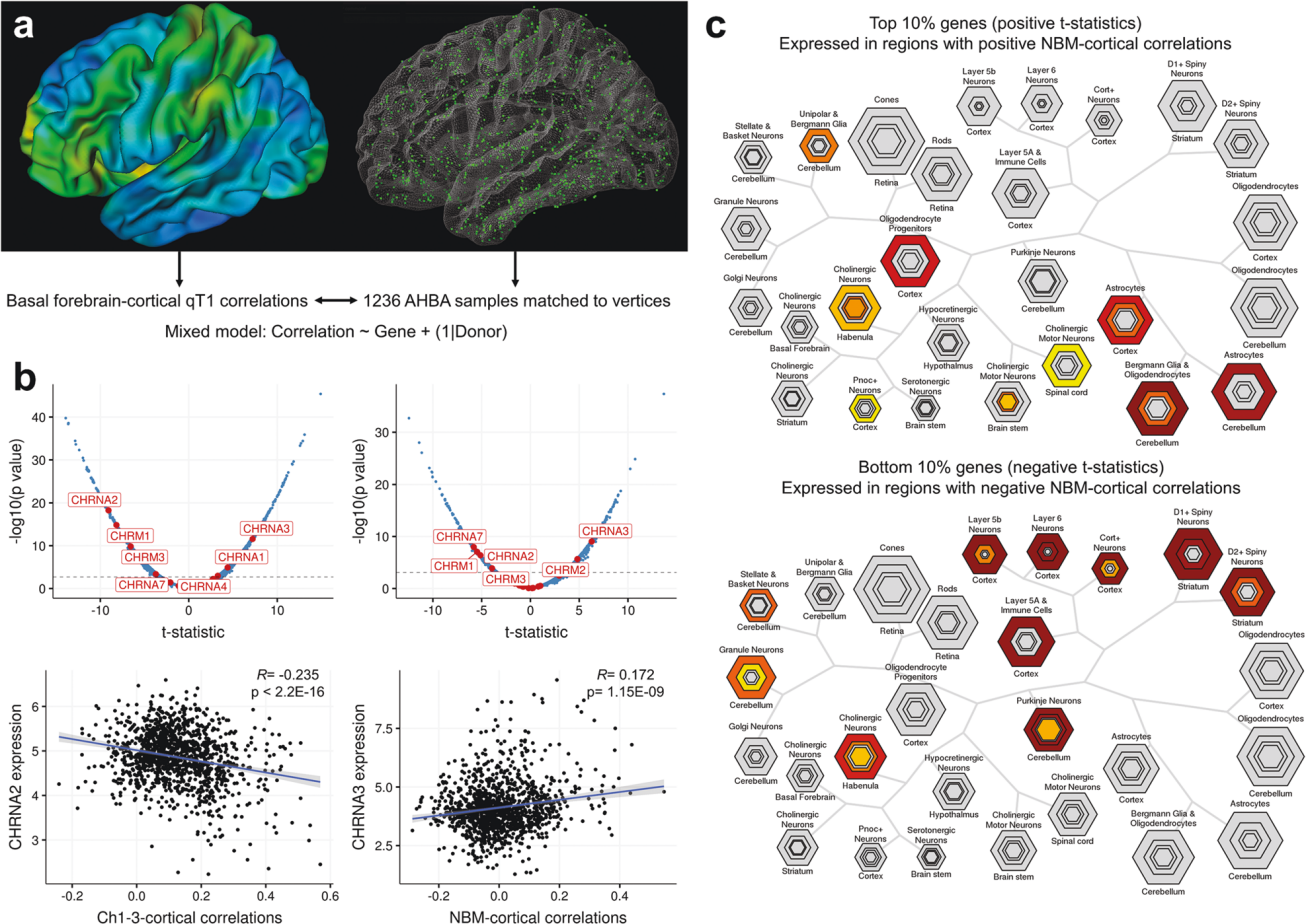
Using transcriptomics to explain neuroimaging correlations. **a** Coloured cortical surface (left) shows Pearson’s R correlations between Ch1-3 qT1 and intracortical qT1. On the right surface, 1236 AHBA cortical samples assigned to a unique vertex are shown. Using a mixed effects model, we sought to explain BF-cortical correlations by gene expression with donor as a random factor. **b** Top: Volcano plot with distribution of t-statistics (*x*-axis), with dotted line indicating significance at FDR *q* < 0.05. Labelled genes are cholinergic receptor genes significant after FDR correction, for the Ch1-3 (left) and NBM (right). Bottom: example of imaging-genetic correlations for 2 of the most significant genes. **c** CSEA of the top 10% (highly expressed in regions with positive NBM-cortical correlations) and bottom 10% genes (highly expressed in regions with negative NBM-cortical correlations) highlights relative enrichment of glial cells in regions with positive imaging correlations.

We sought to explain BF-cortical correlations via gene expression (Fig. 5a). At varying thresholds of FDR (10, 5, 1%), cortical qT1 covariance of NBM was significantly associated with 2262, 1513, and 754 genes respectively, and 4087, 3343, and 2278 genes for Ch1-3-covariance. We found a significant spatial correspondence between BF-cortex qT1 covariance and the distribution of cholinergic receptor gene expression (Fig. 5b), with *CHRNA3* being positively correlated (i.e., highly expressed in those cortical regions with strongest correlations with Ch1-3 and NBM) (Fig. 5b). *CHRNA2* was negatively correlated (i.e., highly expressed in cortical regions with negative correlations with BF) (Fig. 5b). We used CSEA to interpret the imaging-genetic correlations by testing the top and bottom 10% percentile genes (most extreme t-statistics). For NBM-cortical correlations, CSEA of the top 10% (1512) genes (indicating positive t-statistics) shows enrichment for glial cells (astrocytes and oligodendrocytes) and cholinergic neurons, while the bottom 10% shows enrichment for cortical neurons (Fig. 5c). Ch1-3-cortical correlations showed similar results (Supplementary Fig. 3).

### Post hoc analyses

We explored the relationship between cognitive scores (DSST and trail making test), with qT1 and AxD. Pearson’s R correlations were used across 12 tests (2 cognitive scores × 3 regions × 2 imaging modalities) for FEP and HC separately. DSST scores were available for all 29 HC participants and 55 FEP (1 missing) and Trail Making Test for 25 HC (4 missing) and 38 FEP (18 missing). In FEP, there was a significant correlation between left Ch4 qT1 and trail making time (*R* = 0.40, *p* = 0.014, df = 35), and the next strongest correlation was between Ch1-3 AxD and DSST (*R* = 0.27, *p* = 0.053, df = 48). In HC, there was a significant correlation between right Ch4 qT1 and DSST (*R* = 0.45, *p* = 0.013, df = 27). These were not significant after correction for multiple testing (*p*_Bonf_ < 4.17E−03).

Examining RD as an alternative measure for myelin, we found similar findings compared to qT1. Structure-MRS correlations were partly observed with RD: in healthy controls, none of the correlations, without covariates were significant–left NBM was the strongest (*R* = −0.34, *p* = 0.1). After accounting for covariates (age, sex, cannabis use on choline), left NBM correlation was significant (*t* = −4.02, *p* = 7.28E−04)—indicating increased RD (reduced myelin, or myelin damage) predictive of lower choline levels. These correlations were not significant in FEP, with and without accounting for covariates. The diagnosis-by-RD interaction effects were not significant.

In case-control differences of RD, there was a significant difference in Ch1-3 (*t* = 2.264, *p* = 0.028, DF = 50) with increased RD in FEP after taking into account age, sex, cannabis use, and smoking status. This is not significant after multiple testing correction (*p*_Bonf_ < 0.017). Left and right NBM were not significant. Relating clinical scores to RD, left NBM RD was correlated with negative scores (*R* = 0.28, *p* = 0.049, DF = 47), not for YMRS.

## Discussion

**Fig. 6:**
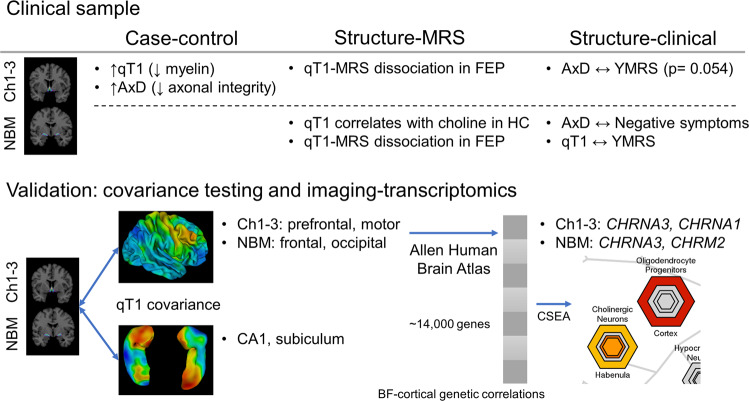
Summary of neuroimaging, clinical, and transcriptomics analyses. (1) Clinical sample neuroimaging results examining case-control differences in microstructure, structure-MRS relationships, and clinical correlates of microstructure. We found increased qT1 and AxD in FEP compared to healthy controls (HC). In HC, there was a significant correlation between qT1 (left NBM) and MRS choline, while left NBM was related to negative (AxD) and manic symptom (qT1) burden. (2) Validation of basal forebrain microstructure measures using qT1 covariance between cortical and subcortical regions, and further external validation of cortical covariance by correlating with gene expression using the Allen Human Brain Atlas. Genes outlined are cholinergic receptor genes most positively associated with BF covariance for Ch1-3 and NBM, surviving FDR correction.

Studying the microstructure of the BF cholinergic nuclei for the first time in first-episode psychosis (FEP), we report an increased qT1 (reflecting reduced myelin content) and increased AxD (reduced axonal integrity) of anteromedial nuclei (Ch1-3) in patients. While dACC choline levels reflected the microstructure of NBM in healthy individuals, this relationship was absent in patients, indicating a possible dissociation between the cholinergic inputs to the Salience Network in psychosis. Our findings (summarised in Fig. 6) agree with the cholinergic-adrenergic hypothesis of mania, and possibly Tandon’s hypothesis that higher cholinergic tone underlies greater negative symptom burden. The transcriptomic analysis of the cholinergic nuclei further supports these findings by highlighting cholinergic neurons and dysfunction in schizophrenia.

While illness-related effects were most pronounced in the anteromedial BF (Ch1-3), the variance in NBM microstructure related to cortical MRS choline, and clinical severity of manic and negative symptoms. This may indicate that a diffuse cholinergic deficit involving hippocampal projections may underlie psychosis, but more extensive deficit involving cortical projections may worsen the symptom burden. This distinction is based only on correlation analyses; studies with longitudinal design are needed to test this speculation. In testing structure-MRS correlations, we found left NBM had stronger correlations to dACC choline than the Ch1-3, which may be due to the NBM containing the highest proportion of cholinergic neurons (at least 90%) compared to Ch1 (10%), Ch2 (70%), Ch3 (1%) in the rhesus monkey [73]. Further, left NBM qT1 and AxD were correlated with manic and negative symptom burden, suggesting the left NBM being most predictive cholinergic function across healthy controls and FEP. As expected, healthy controls, with no perturbation to the BF system, demonstrated a linear association between microstructural integrity and dACC cholinergic levels implying a healthy state. We expected the microstructure of the BF to be perturbed in FEP, resulting in the loss of a linear relationship between dACC choline levels and BF microstructure. This dissociation is likely if cholinergic aberration occurs in a subgroup with aberrant BF microstructure, while the degree of BF aberration per se in most patients with FEP not being sufficiently strong to reduce the absolute concentration of MRS measure of cortical choline.

We used imaging correlations as a proxy for testing the biological validity of qT1 in measuring basal forebrain microstructure. We found significant qT1-based correlations between the BF cholinergic nuclei and cortical (frontal, precentral/postcentral), and subcortical regions (CA1 and subiculum in the hippocampus). Regions with strongest correlations seemingly reflected the known projection patterns from the BF and the presence of cholinergic neurons, in particular the hippocampus with CA1-subiculum [57, 58]. We found significant cortical correlations along the expected medial and lateral pathways from the Ch4 [72], or along the medial cortical surfaces (Fig. 2c). Significant cortical regions including frontal, sensory/motor, and visual cortices are highlighted in previous tracer studies [34, 59–61]. Further biological validation for the imaging correlations are provided by significant associations with cholinergic receptor genes (Fig. 3d) and enrichment for both oligodendrocytes and cholinergic neurons (Fig. 3e). The contrast of the top vs. bottom percentile genes highlights positive enrichment for glial cells, or in other words cortical regions positively correlated with the basal forebrain may house greater density of oligodendrocytes, and overall myelin correlations could be reflective of underlying glia-neuron ratios. Taken together, these analyses strengthen the validity of microstructural measures (qT1, AxD).

In our structural neuroimaging protocol, we found very little grey-white matter contrast in the BF region, therefore, limiting confidence in automated techniques for grey-white matter classification such as VBM, and even less so in measuring its differences across individuals. For example, a recent study with similar methods (using the same cytoarchitectonic atlas) found reduced grey matter integrity, while findings were not significant after accounting for global grey matter volumes [16]—suggesting findings were not region-specific and ascribed to global changes. Here, in the exploration of an alternative approach and using untreated samples, we demonstrated feasibility and biological validity of microstructural measures using both imaging-imaging and imaging-transcriptomic correlations, and results were not impacted by global effects. Our results are further supported by the previous finding of myelin maps as superior in representing cortical circuitry and gene expression compared to other metrics such as cortical thickness [74].

Analysis of basal forebrain gene expression identifies enrichment of schizophrenia genes in both Ch1-3 and NBM, and highlights cholinergic neuron markers (*ACHE, CHAT*). This highlights an understudied component of schizophrenia pathophysiology, and an update building on previous findings [17, 75]. Our results are in line with neuropathological studies, yet there are limited past transcriptomic studies of the BF cholinergic nuclei. While not a focus of the study, we found enrichment of genes associated with major depressive disorder within the BF. As mentioned above, the cholinergic-adrenergic hypothesis of depression [54] along with recent clinical studies has led to renewed interest of the cholinergic system in psychiatric disorders [76, 77]. The biological overlap of cholinergic dysfunction across depression and schizophrenia [78] signals a common dimension that cuts across diagnoses that may explain clinical overlap (such as negative symptoms in schizophrenia that mimic depressive symptoms)—indicating a need to refine our understanding of cholinergic networks to better guide treatment options in psychiatry.

Our study has several strengths as well as limitations. We studied the cholinergic profile of patients experiencing first episode psychosis (mean duration of illness = 31.13 weeks), with minimal or no antipsychotic exposure (lifetime antipsychotic exposure of mean 1.32 DDD equivalents, amounting to < 2 days of exposure to minimal effective doses of antipsychotics) and no exposure to anticholinergic drugs. We quantified nicotine use and adjusted for the observed variance in our analysis. Nevertheless, we could not quantify choline resonance from the BF due to technical limitations of MRS from this anatomical area. There was a limitation in the MRS measures comprising the total choline spectra, which includes choline-containing compounds such as glycerophosphocholine (GPC) and phosphocholine (PC), while the variations in MRS choline may reflect variations in cholinergic tone (discussed in “Introduction”). GPC and PC may be elevated in cases of cell membrane turnover, which may be the case in FEP, yet there were no significant differences in choline between groups. We observed the normative association between BF structure and choline in healthy controls, which was disrupted in FEP—this may be indicative of a subgroup of patients with structural changes and elevated choline (thereby indicating increased cell membrane turnover), contributing to the dissociation of the correlation.

The neuroimaging analyses identified notable findings in the left NBM, but not the right, while the transcriptomic analyses did not allow for separate analysis due to the limited number of samples which could show hemispheric differences. Furthermore, we were limited in the number of female participants in this study; we urge readers to exercise caution when generalising our results to female participants. Lastly, while the majority of the FEP sample (*N* = 56) consists of schizophrenia (*N* = 44, Table 1) and other schizophrenia spectrum disorders (3 schizoaffective, 1 schizophreniform), there are a small number of unclear diagnoses (3 psychosis NOS) and patients later diagnosed with mood disorders (2 MDD, 2 bipolar)—we expect the effects observed to be driven largely by the schizophrenia group, with the limitation that heterogeneity would be expected due to inclusion of all diagnostic groups.

In summary, through a multimodal study employing microstructural imaging, MRS, and indirect inference from transcriptomic correlations, we demonstrate clinical evidence for cholinergic dysfunction in schizophrenia and its relevance to negative and affective symptoms among patients experiencing first-episode of psychosis.

## Supplementary information

Supplementary Materials

## Data Availability

R code used for statistical analysis of neuroimaging and gene expression data are available (https://github.com/mtpark89/NBM_transcriptomic).

## References

[1] Sarter M, Lustig C, Taylor SF (2012). Cholinergic contributions to the cognitive symptoms of schizophrenia and the viability of cholinergic treatments. Neuropharmacology.

[2] Caton M, Ochoa ELM, Barrantes FJ (2020). The role of nicotinic cholinergic neurotransmission in delusional thinking. NPJ Schizophr.

[3] Tandon R (1999). Cholinergic aspects of schizophrenia. Br J Psychiatry Suppl.

[4] Tandon R, Greden JF, Haskett RF (1993). Cholinergic hyperactivity and negative symptoms: Behavioral effects of physostigmine in normal controls. Schizophr Res.

[5] Tandon R, Greden JF, Silk KR (1988). Treatment of negative schizophrenic symptoms with trihexyphenidyl. J Clin Psychopharmacol.

[6] Tandon R, DeQuardo JR, Goodson J, Mann NA, Greden JF (1992). Effect of anticholinergics on positive and negative symptoms in schizophrenia. Psychopharmacol Bull.

[7] Tandon R, Mann NA, Eisner WH, Coppard N (1990). Effect of anticholinergic medication on positive and negative symptoms in medication-free schizophrenic patients. Psychiatry Res.

[8] Shekhar A, Potter WZ, Lightfoot J, Lienemann J, Dubé S, Mallinckrodt C (2008). Selective muscarinic receptor agonist xanomeline as a novel treatment approach for schizophrenia. Am J Psychiatry.

[9] Dean B, Scarr E (2020). Muscarinic M1 and M4 receptors: Hypothesis driven drug development for schizophrenia. Psychiatry Res.

[10] Scarr E, Cowie TF, Kanellakis S, Sundram S, Pantelis C, Dean B (2009). Decreased cortical muscarinic receptors define a subgroup of subjects with schizophrenia. Mol Psychiatry.

[11] Scarr E, Udawela M, Thomas EA, Dean B (2018). Changed gene expression in subjects with schizophrenia and low cortical muscarinic M1 receptors predicts disrupted upstream pathways interacting with that receptor. Mol Psychiatry.

[12] Vingerhoets C, Booij J, van Amelsvoort T Acetylcholine Imaging in Psychosis. In: Dierckx RAJO, Otte A, de Vries EFJ, van Waarde A, Sommer IE, editors. PET and SPECT in psychiatry. Cham: Springer International Publishing; 2021. p. 525–39.

[13] Newton R, Rouleau A, Nylander AG, Loze JY, Resemann HK, Steeves S (2018). Diverse definitions of the early course of schizophrenia-a targeted literature review. NPJ Schizophr.

[14] Liloia D, Brasso C, Cauda F, Mancuso L, Nani A, Manuello J (2021). Updating and characterizing neuroanatomical markers in high-risk subjects, recently diagnosed and chronic patients with schizophrenia: A revised coordinate-based meta-analysis. Neurosci Biobehav Rev.

[15] Gunduz H, Wu H, Ashtari M, Bogerts B, Crandall D, Robinson DG (2002). Basal ganglia volumes in first-episode schizophrenia and healthy comparison subjects. Biol Psychiatry.

[16] Avram M, Grothe MJ, Meinhold L, Leucht C, Leucht S, Borgwardt S, et al. Lower cholinergic basal forebrain volumes link with cognitive difficulties in schizophrenia. Neuropsychopharmacology: Off Publ Am Coll Neuropsychopharmacol. 2021;46:2320–29. 10.1038/s41386-021-01070-x PMC8580980 34188186

[17] Williams MR, Marsh R, Macdonald CD, Jain J, Pearce RK, Hirsch SR (2013). Neuropathological changes in the nucleus basalis in schizophrenia. Eur Arch Psychiatry Clin Neurosci.

[18] Fritz HJ, Ray N, Dyrba M, Sorg C, Teipel S, Grothe MJ (2019). The corticotopic organization of the human basal forebrain as revealed by regionally selective functional connectivity profiles. Hum Brain Mapp.

[19] Braak H, Braak E (1996). Development of Alzheimer-related neurofibrillary changes in the neocortex inversely recapitulates cortical myelogenesis. Acta Neuropathol.

[20] Braak H, Del Tredici K (2004). Poor and protracted myelination as a contributory factor to neurodegenerative disorders. Neurobiol Aging.

[21] Bartzokis G (2007). Acetylcholinesterase inhibitors may improve myelin integrity. Biol Psychiatry.

[22] Stassart RM, Möbius W, Nave KA, Edgar JM (2018). The axon-myelin unit in development and degenerative disease. Front Neurosci.

[23] Zaborszky L, Hoemke L, Mohlberg H, Schleicher A, Amunts K, Zilles K (2008). Stereotaxic probabilistic maps of the magnocellular cell groups in human basal forebrain. NeuroImage.

[24] Sereno MI, Lutti A, Weiskopf N, Dick F (2013). Mapping the human cortical surface by combining quantitative T(1) with retinotopy. Cereb Cortex.

[25] Weiskopf N, Mohammadi S, Lutti A, Callaghan MF (2015). Advances in MRI-based computational neuroanatomy: From morphometry to in-vivo histology. Curr Opin Neurol.

[26] Song SK, Sun SW, Ju WK, Lin SJ, Cross AH, Neufeld AH (2003). Diffusion tensor imaging detects and differentiates axon and myelin degeneration in mouse optic nerve after retinal ischemia. NeuroImage.

[27] Thiessen JD, Zhang Y, Zhang H, Wang L, Buist R, Del Bigio MR (2013). Quantitative MRI and ultrastructural examination of the cuprizone mouse model of demyelination. NMR Biomed.

[28] Lindner M, Bell T, Iqbal S, Mullins PG, Christakou A (2017). In vivo functional neurochemistry of human cortical cholinergic function during visuospatial attention. PLoS One.

[29] Satlin A, Bodick N, Offen WW, Renshaw PF (1997). Brain proton magnetic resonance spectroscopy (1H-MRS) in Alzheimer’s disease: changes after treatment with xanomeline, an M1 selective cholinergic agonist. Am J Psychiatry.

[30] Woo DC, Lenkinski RE (2014). Neurochemical changes observed by in vivo proton magnetic resonance spectroscopy in the mouse brain postadministration of scopolamine. Academic Radiol.

[31] Westman E, Spenger C, Oberg J, Reyer H, Pahnke J, Wahlund LO (2009). In vivo 1H-magnetic resonance spectroscopy can detect metabolic changes in APP/PS1 mice after donepezil treatment. BMC Neurosci.

[32] Wang XC, Du XX, Tian Q, Wang JZ (2008). Correlation between choline signal intensity and acetylcholine level in different brain regions of rat. Neurochem Res.

[33] Bell T, Lindner M, Mullins PG, Christakou A (2018). Functional neurochemical imaging of the human striatal cholinergic system during reversal learning. Eur J Neurosci.

[34] Li X, Yu B, Sun Q, Zhang Y, Ren M, Zhang X (2018). Generation of a whole-brain atlas for the cholinergic system and mesoscopic projectome analysis of basal forebrain cholinergic neurons. Proc Natl Acad Sci USA.

[35] Wu H, Williams J, Nathans J (2014). Complete morphologies of basal forebrain cholinergic neurons in the mouse. eLife.

[36] Kim T, Thankachan S, McKenna JT, McNally JM, Yang C, Choi JH (2015). Cortically projecting basal forebrain parvalbumin neurons regulate cortical gamma band oscillations. Proc Natl Acad Sci USA.

[37] Vingerhoets C, Bakker G, Schrantee A, van der Pluijm M, Bloemen OJN, Reneman L (2019). Influence of muscarinic M(1) receptor antagonism on brain choline levels and functional connectivity in medication-free subjects with psychosis: A placebo controlled, cross-over study. Psychiatry Res Neuroimaging.

[38] Khalighinejad N, Bongioanni A, Verhagen L, Folloni D, Attali D, Aubry JF (2020). A basal forebrain-cingulate circuit in macaques decides it is time to act. Neuron.

[39] Picard F, Sadaghiani S, Leroy C, Courvoisier DS, Maroy R, Bottlaender M (2013). High density of nicotinic receptors in the cingulo-insular network. NeuroImage.

[40] Khalighinejad N, Priestley L, Jbabdi S, Rushworth MFS (2020). Human decisions about when to act originate within a basal forebrain-nigral circuit. Proc Natl Acad Sci USA.

[41] Menon V, Uddin LQ (2010). Saliency, switching, attention and control: A network model of insula function. Brain Struct Funct.

[42] Limongi R, Jeon P, Théberge J, Palaniyappan L. Counteracting effects of glutathione on the glutamate-driven excitation/inhibition imbalance in first-episode schizophrenia: A 7T MRS and dynamic causal modeling study. Antioxidants 2021;10:75. 10.3390/antiox10010075 PMC7828075 33430154

[43] Luo Q, Pan B, Gu H, Simmonite M, Francis S, Liddle PF (2020). Effective connectivity of the right anterior insula in schizophrenia: The salience network and task-negative to task-positive transition. NeuroImage Clin.

[44] Limongi R, Jeon P, Mackinley M, Das T, Dempster K, Théberge J (2020). Glutamate and dysconnection in the salience network: Neurochemical, effective connectivity, and computational evidence in schizophrenia. Biol Psychiatry.

[45] Supekar K, Cai W, Krishnadas R, Palaniyappan L, Menon V (2019). Dysregulated brain dynamics in a triple-network saliency model of schizophrenia and its relation to psychosis. Biol Psychiatry.

[46] Hawrylycz MJ, Lein ES, Guillozet-Bongaarts AL, Shen EH, Ng L, Miller JA (2012). An anatomically comprehensive atlas of the adult human brain transcriptome. Nature.

[47] Howard D, Negraes P, Voineskos AN, Kaplan AS, Muotri AR, Duvvuri V (2020). Molecular neuroanatomy of anorexia nervosa. Sci Rep.

[48] Ibrahim C, Le Foll B, French L (2019). Transcriptomic characterization of the human insular cortex and claustrum. Front Neuroanat.

[49] Mufson EJ, He B, Ginsberg SD, Carper BA, Bieler GS, Crawford F (2018). Gene profiling of nucleus basalis tau containing neurons in chronic traumatic encephalopathy: A chronic effects of neurotrauma consortium study. J Neurotrauma.

[50] Mufson EJ, Counts SE, Ginsberg SD (2002). Gene expression profiles of cholinergic nucleus basalis neurons in Alzheimer’s disease. Neurochem Res.

[51] Seidlitz J, Vasa F, Shinn M, Romero-Garcia R, Whitaker KJ, Vertes PE (2018). Morphometric similarity networks detect microscale cortical organization and predict inter-individual cognitive variation. Neuron.

[52] Tandon R, Taylor SF, DeQuardo JR, Eiser A, Jibson MD, Goldman M (1999). The cholinergic system in schizophrenia reconsidered: Anticholinergic modulation of sleep and symptom profiles. Neuropsychopharmacol: Off Publ Am Coll Neuropsychopharmacol.

[53] Tandon R, Shipley JE, Greden JF, Mann NA, Eisner WH, Goodson JA (1991). Muscarinic cholinergic hyperactivity in schizophrenia. Relationship to positive and negative symptoms. Schizophrenia Res.

[54] Janowsky DS, el-Yousef MK, Davis JM, Sekerke HJ (1972). A cholinergic-adrenergic hypothesis of mania and depression. Lancet.

[55] van Enkhuizen J, Janowsky DS, Olivier B, Minassian A, Perry W, Young JW (2015). The catecholaminergic-cholinergic balance hypothesis of bipolar disorder revisited. Eur J Pharmacol.

[56] Keshavrzi A, Rezaei H, Haghighi M, Jahangard L (2019). Effect of rivastigmine (acetyl cholinesterase inhibitor) versus placebo on manic episodes in patients with bipolar disorders: Results from a double blind, randomized, placebo-controlled clinical trial. Neuropsychobiology.

[57] Frotscher M, Schlander M, Léránth C (1986). Cholinergic neurons in the hippocampus. A combined light- and electron-microscopic immunocytochemical study in the rat. Cell Tissue Res.

[58] Agostinelli LJ, Geerling JC, Scammell TE (2019). Basal forebrain subcortical projections. Brain Struct Funct.

[59] Chandler D, Waterhouse BD (2012). Evidence for broad versus segregated projections from cholinergic and noradrenergic nuclei to functionally and anatomically discrete subregions of prefrontal cortex. Front Behav Neurosci.

[60] Chandler DJ, Lamperski CS, Waterhouse BD (2013). Identification and distribution of projections from monoaminergic and cholinergic nuclei to functionally differentiated subregions of prefrontal cortex. Brain Res.

[61] Bloem B, Schoppink L, Rotaru DC, Faiz A, Hendriks P, Mansvelder HD (2014). Topographic mapping between basal forebrain cholinergic neurons and the medial prefrontal cortex in mice. J Neurosci: Off J Soc Neurosci.

[62] Dempster K, Jeon P, MacKinley M, Williamson P, Théberge J, Palaniyappan L (2020). Early treatment response in first episode psychosis: A 7-T magnetic resonance spectroscopic study of glutathione and glutamate. Mol Psychiatry.

[63] Park MTM, Jeon P, Khan AR, Dempster K, Chakravarty MM, Lerch JP (2021). Hippocampal neuroanatomy in first episode psychosis: A putative role for glutamate and serotonin receptors. Prog Neuro-Psychopharmacol Biol Psychiatry.

[64] Pan Y, Dempster K, Jeon P, Théberge J, Khan AR, Palaniyappan L (2021). Acute conceptual disorganization in untreated first-episode psychosis: A combined magnetic resonance spectroscopy and diffusion imaging study of the cingulum. J Psychiatry Neurosci: JPN.

[65] Avants BB, Epstein CL, Grossman M, Gee JC (2008). Symmetric diffeomorphic image registration with cross-correlation: Evaluating automated labeling of elderly and neurodegenerative brain. Med Image Anal.

[66] Miller JA, Menon V, Goldy J, Kaykas A, Lee CK, Smith KA (2014). Improving reliability and absolute quantification of human brain microarray data by filtering and scaling probes using RNA-Seq. BMC Genomics.

[67] Le Foll B, French L (2018). Transcriptomic characterization of the human habenula highlights drug metabolism and the neuroimmune system. Front Neurosci.

[68] Benjamini Y, Hochberg Y (1995). Controlling the false discovery rate: A practical and powerful approach to multiple testing. J R Stat Soc Ser B (Methodol).

[69] Dougherty JD, Schmidt EF, Nakajima M, Heintz N (2010). Analytical approaches to RNA profiling data for the identification of genes enriched in specific cells. Nucleic Acids Res.

[70] Xu X, Wells AB, O’Brien DR, Nehorai A, Dougherty JD (2014). Cell type-specific expression analysis to identify putative cellular mechanisms for neurogenetic disorders. J Neurosci: Off J Soc Neurosci.

[71] Chen J, Bardes EE, Aronow BJ, Jegga AG (2009). ToppGene Suite for gene list enrichment analysis and candidate gene prioritization. Nucleic Acids Res.

[72] Mesulam MM (2013). Cholinergic circuitry of the human nucleus basalis and its fate in Alzheimer’s disease. J Comp Neurol.

[73] Mesulam MM, Mufson EJ, Levey AI, Wainer BH (1983). Cholinergic innervation of cortex by the basal forebrain: Cytochemistry and cortical connections of the septal area, diagonal band nuclei, nucleus basalis (substantia innominata), and hypothalamus in the rhesus monkey. J Comp Neurol.

[74] Burt JB, Demirtaş M, Eckner WJ, Navejar NM, Ji JL, Martin WJ (2018). Hierarchy of transcriptomic specialization across human cortex captured by structural neuroimaging topography. Nat Neurosci.

[75] Holt DJ, Bachus SE, Hyde TM, Wittie M, Herman MM, Vangel M (2005). Reduced density of cholinergic interneurons in the ventral striatum in schizophrenia: An in situ hybridization study. Biol Psychiatry.

[76] Dulawa SC, Janowsky DS (2019). Cholinergic regulation of mood: from basic and clinical studies to emerging therapeutics. Mol Psychiatry.

[77] Joshi YB, Thomas ML, Braff DL, Green MF, Gur RC, Gur RE, et al. Anticholinergic medication burden-associated cognitive impairment in schizophrenia. Am. J Psychiatry. 2021. 10.1176/appi.ajp.2020.20081212. 10.1176/appi.ajp.2020.20081212 PMC8440496 33985348

[78] Higley MJ, Picciotto MR (2014). Neuromodulation by acetylcholine: Examples from schizophrenia and depression. Curr Opin Neurobiol.

