# Supplementary material for: Microstructural imaging and transcriptomics of the basal forebrain in first-episode psychosis
Source: Transl Psychiatry. 2022 Sep 1;12:358. doi: 10.1038/s41398-022-02136-0 (PMC9436926; doi:10.1038/s41398-022-02136-0)
Supplement: Supplementary file 1 — Supplementary Materials [file 41398_2022_2136_MOESM1_ESM.docx]

**Supplementary Information: Myelin imaging of the basal forebrain in first-episode psychosis**

Min Tae M. Park^1^, Peter Jeon^2^, Leon French^3^, Kara Dempster^4^, Julie Richard^1^, M. Mallar Chakravarty^5,6^, Jean Théberge^1,7,8^, Ali R. Khan^2,8^, Lena Palaniyappan^1,2,7,8^

1. Supplementary Methods
2. Supplementary Figures

**Supplementary Methods**

**Acquisition of neuroimaging and preprocessing.** We employed a Siemens MAGNETOM 7.0T MRI Plus (Siemens, Erlangen, Germany) using an 8-channel transmit/32-channel receive, head-only, radiofrequency coil at the Centre for Functional and Metabolic Mapping at Western University in London, Ontario. Anatomical images of each participant were acquired using a sagittal 3D MP2RAGE^1^ sequence with TE=2.83 ms, TR=6000 ms, TI=800ms/2700 ms, flip angle=4/5 degrees, matrix=320x320x208, iPAT=3, partial Fourier=6/8, voxel size=0.8x0.8x0.8,  and a 3D SA2RAGE^2^, with TE=0.81 ms, TR=2400 ms, TI=45/1800 ms, flip angle=4^o^/5^o^, matrix=320x320x208, iPAT=3, partial Fourier=6/8, voxel size=0.8mmx0.8mmx0.8mm.  Images were first corrected for gradient non-linearities using spherical harmonic coefficients provided by the manufacturer in addition to a spline interpolation and modulation of the deformation field as implemented in a BIDS^3^ processing pipeline (gradcorrect, https://github.com/khanlab/gradcorrect). The T1 maps and T1-weighted images (MP2RAGE UNI-DEN images) were then corrected for residual B1+ non-uniformities by alternating estimation of T1 and B1 maps using a lookup table approach^1^, as implemented in a BIDS app (https://github.com/khanlab/mp2rage_correct). Brain extraction was performed on these corrected UNI-DEN images using the multi-template BEaST approach, with a customized template library based on 19 scans (38 after left-right flipping) manually corrected for accuracy as recommended^4^. Intracranial volume (ICV) is computed using the jacobian determinant of the linear registration to common space used in BEaST.

**Diffusion tensor imaging.** Diffusion tensor images were acquired using 64 diffusion-weighted volumes (b value = 1000 s/mm^2^) and an unweighted volume, with a 2-dimensional, spin-echo, echo-planar imaging sequence (matrix size = 104 × 104; 72 slices; voxel resolution 2 mm × 2 mm × 2 mm; repetition time 5100 ms; echo time 50.2 ms; generalized autocalibrating partial parallel acquisition 3; multi-band 3; phase partial Fourier 6/8). We processed diffusion-weighted images using an open-source, containerized application called “prepdwi” (www.github.com/khanlab/prepdwi), which uses the BIDS^5^ and BIDS Apps^3^ standards for pre-processing and fitting.

In line with our prior work^6^, we first preprocessed diffusion-weighted MRI data with denoising using a local PCA method with “dwidenoise” from mrtrix^7^ and correction of ringing artifacts with the “unring” tool^8^. We corrected eddy current and susceptibility-based distortions using “topup”^9^ and “eddy”^10^ based on FSL with the “--repol” option enabled for outlier replacement^11^. DWI images were registered to T1w space (within-subject) using block-matching^12^. Gradient non-linearities were corrected using vendor-provided spherical harmonic coefficients for the gradient system (AC84), applied with the “gradient_unwarp” tool^13^ to generate a nonlinear transformation, which was composed with the T1-weighted linear transformation to resample the DWI images into the corrected T1-weighted space in a single step. Modulation with the determinant of the Jacobian of the unwarping was used to correct for intensity differences in the magnitude images due to gradient non-linearities. Pre-processed DWI images in the T1w space were then used to estimate diffusion tensor metrics using “dtifit”^14^. We used the derived AxD and radial diffusivity (RD) maps to measure mean AxD and RD of BF structures.

**MRS data acquisition.** A 2.0 cm x 2.0 cm x 2.0 cm (8 cm^3^) 1H-MRS voxel were placed in the bilateral dorsal ACC (dACC) using a two-dimensional anatomical imaging sequence in the sagittal direction (37 slices, TR=8000ms, TE=70ms, flip-angle=120°, thickness= 3.5mm, field of view= 240×191mm). Voxel positioning is described in our previous work^15^—briefly, the posterior end of the voxel was set to coincide with the precentral gyrus, while the caudal face of the voxel coinciding with the most caudal positioning that was not part of the corpus callosum. Voxel angle was tangential to the corpus callosum.

A total of 32 channel-combined, water-suppressed spectral averages were acquired using the semi-LASER 1H-MRS pulse sequence (TR=7500ms, TE=100ms) during each scan session, while participants were at rest and asked to keep their gaze fixated on a white cross against a 50% gray background projected on a screen for 4 minutes. Water suppression was achieved using the VAPOR preparation sequence, and water-unsuppressed spectra were acquired for lineshape deconvolution and spectral quantification. Each of the 32 spectral acquisitions was corrected for frequency and phase drifts as described in Near et al^16^ prior to averaging. Further spectral post-processing (QUECC^17^ and HSVD^18^ water removal) and spectral fitting were done using fitMAN^19^, a time-domain fitting algorithm that uses a nonlinear, iterative Levenberg-Marquardt minimization algorithm to estimate the chemical shift, amplitude, linewidth and phase of echo time-specific prior knowledge templates. A total of 17 brain metabolites were included in the fitting template as described in our previous work^20^. Metabolite concentrations were quantified, using the Barstool software^21^, after correcting for gray and white matter volumes. Quality of all spectra was inspected visually and only metabolites quantified with Cramer-Rao lower bounds (CRLB) lower than 10% were included in the analyses.

**Cortical and subcortical qT1 mapping.** High-resolution T1 images were used to map the cortex using the CIVET pipeline version 2.1.0. Intracortical qT1 values were sampled at 50% depth between the pial and white matter boundary and blurred using a 20mm kernel. MAGeT Brain pipeline generated subcortical surfaces (hippocampus, amygdala, striatum, thalamus, globus pallidum)^22, 23^ were used to sample vertex-wise qT1 values along all surfaces and smoothed using a 5mm kernel (2mm for globus pallidum) ^24-27^. All CIVET surfaces and subcortical segmentations were visually expected for quality, and only those passing quality control were included in relevant analysis.

**Neuroimaging of the basal forebrain.** We used a published probabilistic atlas^28^ of the BF from a cytoarchitectonic mapping of cholinergic cell groups defined as Ch1-3 (septum and diagonal band) and Ch4 (NBM). The atlas has been previously employed in several neuroimaging studies^29-32^. Probabilistic maps were generated from histological sections from 10 post-mortem brains, and mapped to the Colin27 template^33^, available within the SPM Anatomy Toolbox (https://www.fil.ion.ucl.ac.uk/spm/ext/). ANTS^34^ linear and non-linear registrations were used to transform the Colin27 brain onto individual T1 images. To increase registration accuracy, 3 separate registrations are performed for the Ch1-3, left and right NBM via an automated masking procedure (https://github.com/CoBrALab/MAGeTbrain) to focus on a given ROI. Transformed probabilistic maps (in subject space) were thresholded at 0.50 (voxels identified as basal forebrain in at least 50% of the brains in the original paper). Using these subject-specific labels of the BF structures, we obtained the mean qT1 and AxD of each for all participants. Probabilistic maps were visually inspected for accuracy, excluding those with poor registrations.

**
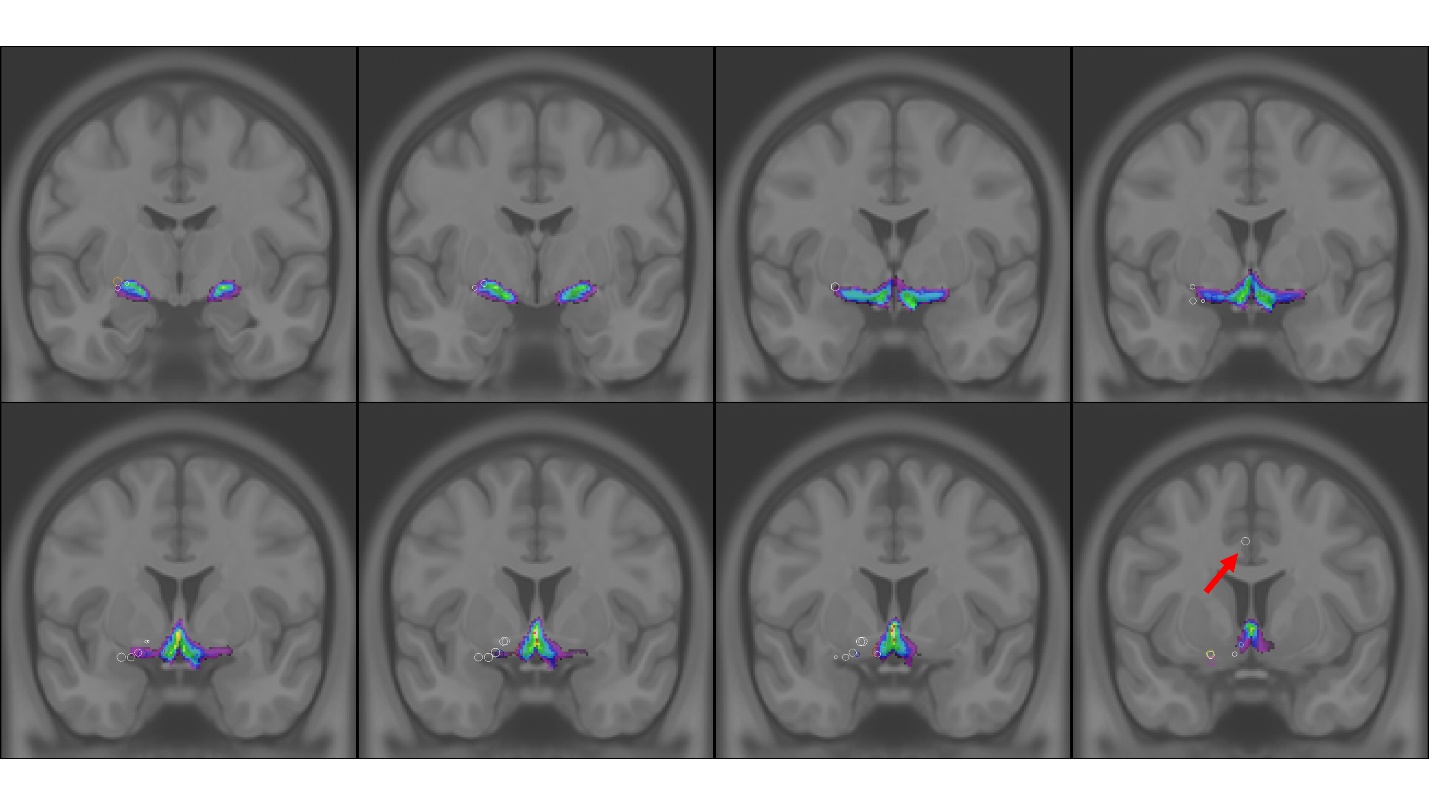
Supplementary Figure 1.** Visual quality control of basal forebrain samples from Allen Human Brain Atlas, projected onto the MNI template. Coloured regions (spectral) show probabilistic atlas transformed to the MNI template, while circles indicate locations of Allen Brain samples. One Ch1-3 sample was misplaced or mislabelled (red arrow) and excluded from analysis.


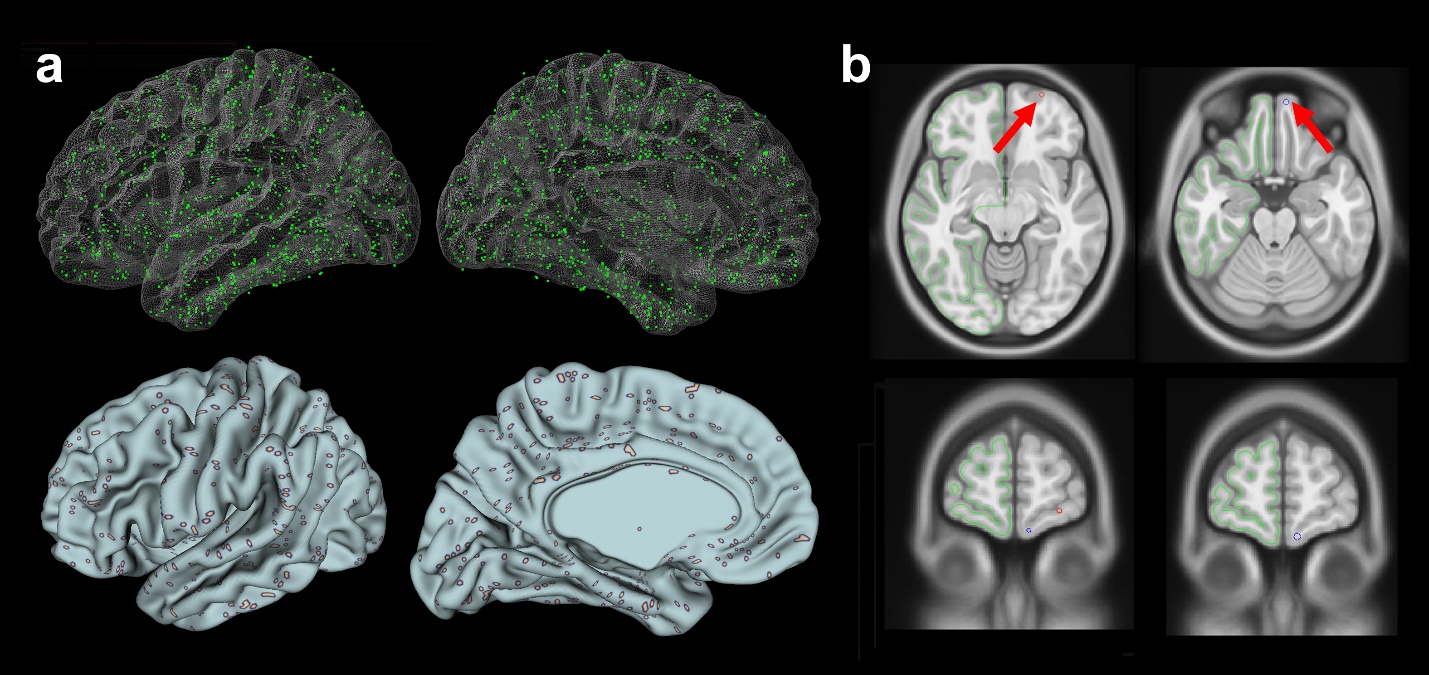
**Supplementary Figure 2.** Matching AHBA samples to CIVET cortical vertices for the left hemisphere. a) Top: green points show location of 1,236 AHBA cortical samples, bottom: matching unique vertices on the cortical surface are coloured. b) Visual quality control of left cortical samples from Allen Human Brain Atlas, projected onto the MNI template. 2 samples (red arrows) had distance greater than 10mm to the nearest cortical vertex, found in the right hemisphere, and removed from the analysis.

**
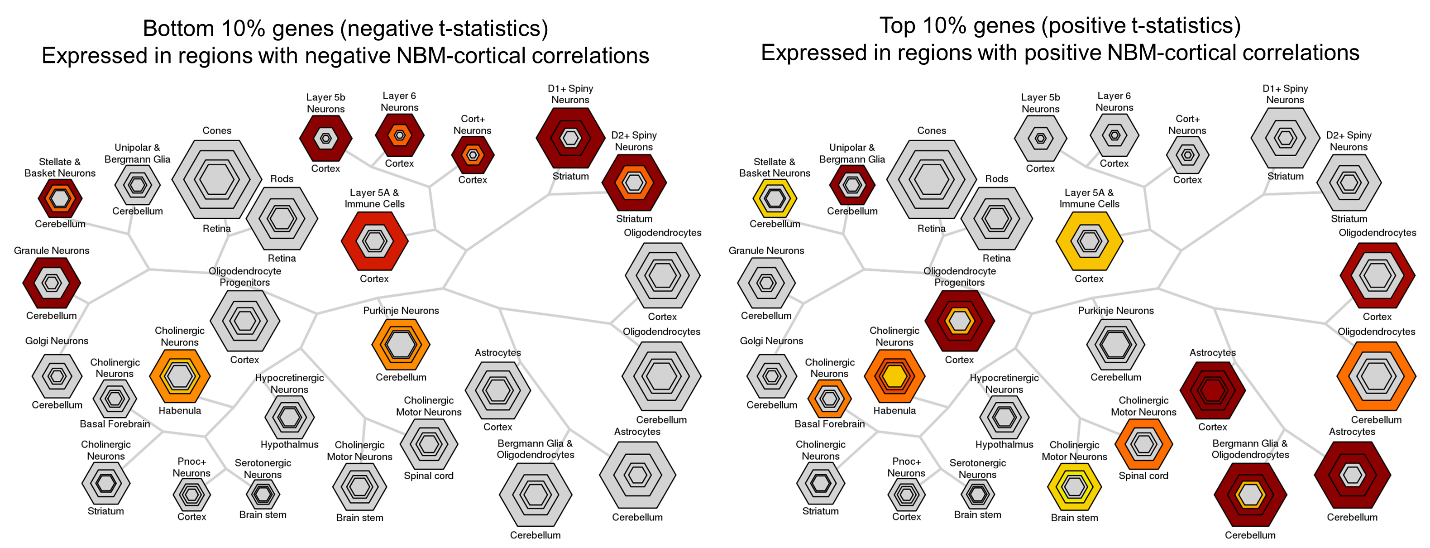
Supplementary Figure 3.** CSEA of the top 10% (highly expressed in regions with positive Ch1-3-cortical correlations) and bottom 10% genes (highly expressed in regions with negative Ch1-3-cortical correlations) highlights relative enrichment of glial cells in regions with positive imaging correlations.

**References**

1. Marques JP, Kober T, Krueger G, van der Zwaag W, Van de Moortele PF, Gruetter R. MP2RAGE, a self bias-field corrected sequence for improved segmentation and T1-mapping at high field. *NeuroImage* 2010; **49**(2)**:** 1271-1281.

2. Eggenschwiler F, Kober T, Magill AW, Gruetter R, Marques JP. SA2RAGE: a new sequence for fast B1+ -mapping. *Magnetic resonance in medicine : official journal of the Society of Magnetic Resonance in Medicine / Society of Magnetic Resonance in Medicine* 2012; **67**(6)**:** 1609-1619.

3. Gorgolewski KJ, Alfaro-Almagro F, Auer T, Bellec P, Capota M, Chakravarty MM *et al.* BIDS apps: Improving ease of use, accessibility, and reproducibility of neuroimaging data analysis methods. *Plos Computational Biology* 2017; **13**(3).

4. Eskildsen SF, Coupe P, Fonov V, Manjon JV, Leung KK, Guizard N *et al.* BEaST: brain extraction based on nonlocal segmentation technique. *NeuroImage* 2012; **59**(3)**:** 2362-2373.

5. Gorgolewski KJ, Auer T, Calhoun VD, Craddock RC, Das S, Duff EP *et al.* The brain imaging data structure, a format for organizing and describing outputs of neuroimaging experiments. *Sci Data* 2016; **3**.

6. Pan Y, Dempster K, Jeon P, Théberge J, Khan AR, Palaniyappan L. Acute conceptual disorganization in untreated first-episode psychosis: a combined magnetic resonance spectroscopy and diffusion imaging study of the cingulum. *Journal of psychiatry & neuroscience : JPN* 2021; **46**(3)**:** E337-e346.

7. Veraart J, Novikov DS, Christiaens D, Ades-Aron B, Sijbers J, Fieremans E. Denoising of diffusion MRI using random matrix theory. *Neuroimage* 2016; **142:** 384-396.

8. Kellner E, Dhital B, Kiselev VG, Reisert M. Gibbs-Ringing Artifact Removal Based on Local Subvoxel-Shifts. *Magnetic Resonance In Medicine* 2016; **76**(5)**:** 1574-1581.

9. Andersson JLR, Skare S, Ashburner J. How to correct susceptibility distortions in spin-echo echo-planar images: application to diffusion tensor imaging. *Neuroimage* 2003; **20**(2)**:** 870-888.

10. Andersson JLR, Sotiropoulos SN. An integrated approach to correction for off-resonance effects and subject movement in diffusion MR imaging. *Neuroimage* 2016; **125:** 1063-1078.

11. Andersson JLR, Graham MS, Zsoldos E, Sotiropoulos SN. Incorporating outlier detection and replacement into a non-parametric framework for movement and distortion correction of diffusion MR images. *Neuroimage* 2016; **141:** 556-572.

12. Modat M, Cash D, Daga P, Winston G, Duncan J, Ourselin S. Global image registration using a symmetric block-matching approach. *Journal of medical imaging (Bellingham, Wash)* 2014; **1**(2)**:** 024003.

13. Jovicich J, Czanner S, Greve D, Haley E, van der Kouwe A, Gollub R *et al.* Reliability in multi-site structural MRI studies: Effects of gradient non-linearity correction on phantom and human data. *Neuroimage* 2006; **30**(2)**:** 436-443.

14. Jenkinson M, Beckmann CF, Behrens TE, Woolrich MW, Smith SM. Fsl. *Neuroimage* 2012; **62**(2)**:** 782-790.

15. Dempster K, Jeon P, MacKinley M, Williamson P, Théberge J, Palaniyappan L. Early treatment response in first episode psychosis: a 7-T magnetic resonance spectroscopic study of glutathione and glutamate. *Molecular psychiatry* 2020; **25**(8)**:** 1640-1650.

16. Near J, Edden R, Evans CJ, Paquin R, Harris A, Jezzard P. Frequency and phase drift correction of magnetic resonance spectroscopy data by spectral registration in the time domain. *Magnetic resonance in medicine : official journal of the Society of Magnetic Resonance in Medicine / Society of Magnetic Resonance in Medicine* 2015; **73**(1)**:** 44-50.

17. Bartha R, Drost DJ, Menon RS, Williamson PC. Spectroscopic lineshape correction by QUECC: combined QUALITY deconvolution and eddy current correction. *Magnetic resonance in medicine : official journal of the Society of Magnetic Resonance in Medicine / Society of Magnetic Resonance in Medicine* 2000; **44**(4)**:** 641-645.

18. van den Boogaart A, Ala-Korpela M, Jokisaari J, Griffiths JR. Time and frequency domain analysis of NMR data compared: an application to 1D 1H spectra of lipoproteins. *Magnetic resonance in medicine : official journal of the Society of Magnetic Resonance in Medicine / Society of Magnetic Resonance in Medicine* 1994; **31**(4)**:** 347-358.

19. Bartha R, Drost DJ, Williamson PC. Factors affecting the quantification of short echo in-vivo 1H MR spectra: prior knowledge, peak elimination, and filtering. *NMR in biomedicine* 1999; **12**(4)**:** 205-216.

20. Jeon P, Limongi R, Ford SD, Mackinley M, Dempster K, Théberge J *et al.* Progressive Changes in Glutamate Concentration in Early Stages of Schizophrenia: A Longitudinal 7-Tesla MRS Study. *Schizophr Bull Open* 2021; **2**(1)**:** sgaa072.

21. Wong D, Schranz AL, Bartha R. Optimized in vivo brain glutamate measurement using long-echo-time semi-LASER at 7 T. *NMR in biomedicine* 2018; **31**(11)**:** e4002.

22. Tullo S, Devenyi GA, Patel R, Park MTM, Collins DL, Chakravarty MM. Warping an atlas derived from serial histology to 5 high-resolution MRIs. *Sci Data* 2018; **5:** 180107.

23. Winterburn JL, Pruessner JC, Chavez S, Schira MM, Lobaugh NJ, Voineskos AN *et al.* A novel in vivo atlas of human hippocampal subfields using high-resolution 3 T magnetic resonance imaging. *NeuroImage* 2013; **74:** 254-265.

24. Chakravarty MM, Steadman P, van Eede MC, Calcott RD, Gu V, Shaw P *et al.* Performing label-fusion-based segmentation using multiple automatically generated templates. *Human brain mapping* 2013; **34**(10)**:** 2635-2654.

25. Raznahan A, Shaw PW, Lerch JP, Clasen LS, Greenstein D, Berman R *et al.* Longitudinal four-dimensional mapping of subcortical anatomy in human development. *Proceedings of the National Academy of Sciences of the United States of America* 2014; **111**(4)**:** 1592-1597.

26. Chakravarty MM, Rapoport JL, Giedd JN, Raznahan A, Shaw P, Collins DL *et al.* Striatal shape abnormalities as novel neurodevelopmental endophenotypes in schizophrenia: A longitudinal study. *Human brain mapping* 2014.

27. Pipitone J, Park MT, Winterburn J, Lett TA, Lerch JP, Pruessner JC *et al.* Multi-atlas segmentation of the whole hippocampus and subfields using multiple automatically generated templates. *NeuroImage* 2014; **101:** 494-512.

28. Zaborszky L, Hoemke L, Mohlberg H, Schleicher A, Amunts K, Zilles K. Stereotaxic probabilistic maps of the magnocellular cell groups in human basal forebrain. *NeuroImage* 2008; **42**(3)**:** 1127-1141.

29. Schmitz TW, Nathan Spreng R. Basal forebrain degeneration precedes and predicts the cortical spread of Alzheimer's pathology. *Nature communications* 2016; **7:** 13249.

30. Schmitz TW, Mur M, Aghourian M, Bedard MA, Spreng RN. Longitudinal Alzheimer's Degeneration Reflects the Spatial Topography of Cholinergic Basal Forebrain Projections. *Cell Rep* 2018; **24**(1)**:** 38-46.

31. Markello RD, Spreng RN, Luh WM, Anderson AK, De Rosa E. Segregation of the human basal forebrain using resting state functional MRI. *NeuroImage* 2018; **173:** 287-297.

32. Lammers F, Mobascher A, Musso F, Shah NJ, Warbrick T, Zaborszky L *et al.* Effects of Ncl. Basalis Meynert volume on the Trail-Making-Test are restricted to the left hemisphere. *Brain and behavior* 2016; **6**(1)**:** e00421.

33. Holmes CJ, Hoge R, Collins L, Woods R, Toga AW, Evans AC. Enhancement of MR images using registration for signal averaging. *Journal of computer assisted tomography* 1998; **22**(2)**:** 324-333.

34. Avants BB, Epstein CL, Grossman M, Gee JC. Symmetric diffeomorphic image registration with cross-correlation: evaluating automated labeling of elderly and neurodegenerative brain. *Medical image analysis* 2008; **12**(1)**:** 26-41.
